# Fall prevention through a systems approach: SEIPS, AcciMap and HTA findings from patient safety specialists

**DOI:** 10.1093/ageing/afaf374

**Published:** 2026-01-08

**Authors:** Mike Fray, Gyuchan Thomas Jun, Sue Hignett

**Affiliations:** School of Design & Creative Arts, Loughborough University, Loughborough, UK; School of Design & Creative Arts, Loughborough University, Loughborough, UK; School of Design & Creative Arts, Loughborough University, Loughborough, UK

**Keywords:** systems thinking, human factors, Hierarchy of Controls, falls prevention, older people

## Abstract

In 2022, two reviews were published in Age & Ageing to summarise and discuss falls prevention interventions. One was a narrative review looking at the past, present and future of falls interventions. The second was a systematic review and meta-analysis of research on adult falls in hospital. This commentary discusses the findings and recommendations of these publications in the context of real world practice using the Hierarchy of Controls framework and case studies from 180 Patient Safety Specialists applying three systems tools and approaches; Systems Engineering Initiative for Patient Safety, Hierarchical Task Analysis and AcciMaps. The reflection on research (published reviews) and practice (case studies) offers an insight for interventions in practice, rather than research. We hope that future researchers and funders in falls prevention will embrace safety science tools/approaches when planning and reviewing project proposals.

## Key Points

- Transfer of falls research findings into practice has been limited by investment and scale-up challenges
- Hierarchy of Controls (HoC) provides a reflective framework to consider likely effectiveness of interventions
- Most previous falls interventions have been at the least effective levels of the HoC (administrative and PPE)
- Taking a Systems approach in practice with Safety science tools (Systems Engineering Initiative for Patient Safety, Hierarchical Task Analysis, AcciMap) supports a change in falls prevention
- Future falls prevention research should embed safety science tools and approaches

## Introduction

In 2022, two reviews were published in Age & Ageing to summarise and discuss falls prevention interventions. Close and Lord [1] provided a narrative overview of interventions from single modality (e.g. exercise, podiatry, vitamin D supplements) and multifactorial (including new technologies) intervention research. They reflected that ‘many of the trials have been time and funding limited and there has been insufficient investment in implementation and scale-up of interventions’, suggesting that the transfer of research into practice is limited. Morris *et al*. [2] reported a systematic review and meta-analysis for fall prevention interventions for hospitalised adults. They concluded that ‘Patient and staff education can reduce hospital falls. Multifactorial interventions had a tendency towards producing a positive impact. Chair alarms, bed alarms, wearable sensors and use of scored risk assessment tools were not associated with significant fall reductions’. This provides a clear platform to take a systems approach (multifactorial) for fall prevention.

In this commentary, we will reflect on these reviews using the framework of the Hierarchy of Controls (HoC) and case study outputs (*n* = 180) from a patient safety training programme in England where Falls incidents were analysed using safety science tools.

## Falls prevention in practice: systems thinking

In 2019, a new Patient Safety Strategy [3] was launched in England, followed with the Patient Safety Syllabus [4] in 2022, to change how the National Health Service (NHS) thinks and acts about patient safety. The syllabus has 5 levels, with Levels 1 and 2 available for all NHS staff as an eLearning resource. Levels 3 and 4 offer more detailed training and practice to support specialist staff.

In 2023, a national tender was issued to provide Levels 3 and 4 to Patient Safety Specialists (PSS) for acute, ambulance, mental health, community providers, and regional and national advisory services. This was delivered as a blended learning programme with five online courses (A–E), five in-person days and six assessed case studies. Over 950 PSS were contacted, with over 650 enrolling and 483 completing.

**Figure 1:**
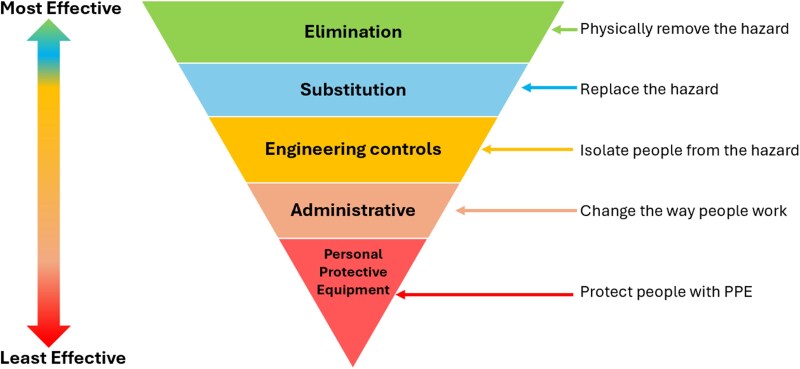
Hierarchy of Controls (modified from NIOSH [5]).

Throughout the syllabus delivery, the PSS were encouraged to think about the effectiveness of safety controls using the framework of the Hierarchy of Controls [5] (HoC; Figure 1) to identify a preferred order of actions. Elimination, substitution and engineering controls are described as more effective actions because they control exposures without significant human interaction, whereas administrative (training, procedures/policies) and personal protective equipment (modifying the human) are considered to be the least effective safety actions. This HoC framework has been discussed for the healthcare context [6] and it has been suggested that currently most healthcare safety and risk management actions are at the least effective levels [7].

The case study topic for Course A was to apply safety science tools to any aspect of Falls (patients, staff, visitors etc.). Each submission was marked, and a subset were moderated to agree boundaries (pass/resubmit, pass/excellent). The PSSs used a provided template with sections for context/background, author’s role, summary, outputs and future ideas, and reflections, with a word count maximum of 1000 words. All included the following permission statement ‘I consent to the re-use of anonymized data for future research not directly linked to this Case Study but for improving healthcare safety’.

The largest group of case studies (89%) described falls related to the care of older people in different settings including acute hospitals, mental health, community, care homes and ambulance transport. In some cases, the PSS were directly involved in the incident and in others, they were involved in the investigation process/review.

The included case studies were marked as excellent (*n* = 180, 35% of submissions) and used two or more of the Course A tools, SEIPS (Systems Engineering Initiative for Patient Safety), AcciMap and HTA (Hierarchical Task Analysis):

- SEIPS is a framework used to analyse complex systems and understand how different factors interact to produce outcomes [8]. This was used with AcciMap (*n* = 88) and with HTA (*n* = 80).
- AcciMap is an accident analysis technique used to map contributory factors as interrelationships between different levels within a complex socio-technical system [9]. This was used as above, and with HTA (*n* = 5).
- HTA describes a task as a hierarchy of superordinate and subordinate tasks as a way of stating how work should be organised to meet system goals. It is useful for visualising and comparing work-as-done with work-as-imagined (procedures etc.) [10]. This was used as above, and with both SEIPS and AcciMap (*n* = 10).

The outputs and future ideas section for each case study was exported in NVivo for Thematic analysis. The data were coded by action type, the codes iteratively explored and the outputs outlined as follows.

## Likely effectiveness of interventions: Hierarchy of Controls

The most mentioned approach was administrative, with changes proposed to policies and procedures, for example adding recommendations into the 999 (emergency) call script [*PSS 164*], creating a flowchart for monitoring a patient [*PSS 509*], educating staff and families on the importance of safety protocols and compliance with the relevant policies [*PSS 654*] and linking medical devices policy with falls policy [*PSS 1030*]. All useful actions, but we suggest that on the HoC framework, these actions are over-reliant on human behaviour and humans as ‘successful adapters’ to ‘cope with complexities, uncertainties, and goal conflicts of complex work settings’ [11]. This perhaps reflects the relative immaturity of this new safety workforce [12] and the early stage of PSS training (Course A); by Course E the use of multiple safety science tools in case studies had increased from 35% to 68%.

## Patient safety specialists’ reflections

The final part of the case study asked the PSS to reflect ‘How has this case study changed your approach to healthcare safety?’ The following three examples offer some insight for changes in thinking:

- Falls are ‘not just an elderly care problem … thoughts immediately turn to modification of patient or drug-related risk factors; these are the factors that clinicians most commonly deal with. However, we sometimes have little control over patient risk factors, therefore viewing the environment as a stable factor that we can control offers the opportunity for more permanent changes [PSS 1229]’
- ‘While gathering a multidisciplinary team to plot the incident took time and effort, the SEIPS and AcciMap tools brought a new perspective. This case study showed us that by looking at things “in the round” and outside of traditional root cause analysis, we see a much bigger picture. This enables us to improve at a system level, rather than just put right the things that went wrong. We will be adopting these tools going forward’. [PSS 888]
- ‘It is absolutely imperative to understand “work as done” versus “work as imagined” and to engage staff fully in the improvement/investigative process to help support a more meaningful outcome and ensure improvements are embedded in everyday practice. It also ensures that staff feel both engaged and more importantly feel valued. The SEIPS tool and Accimap really helped with the understanding between relationships, patient complexities, the environment, interaction of processes and the organisational structure’. [PSS 1286].

## Future falls prevention research should embed safety science tools and approaches

The reflection on research (published reviews) and practice (case studies) offers food for thought and provides an interesting insight for interventions in practice, rather than research. We are hopeful that the use of safety science tools [13] to support systems thinking (and multifactorial interventions) will embed as an outcome from this national training programme. There are applications for systems thinking tools and approaches in many areas, including clinical work design, risk assessment, quality improvement projects and investigation processes. We hope that future researchers and funders in falls prevention will also embrace safety science tools/approaches when planning and reviewing project proposals.
